# Multi-day dataset of forearm and wrist electromyogram for hand gesture recognition and biometrics

**DOI:** 10.1038/s41597-022-01836-y

**Published:** 2022-11-30

**Authors:** Ashirbad Pradhan, Jiayuan He, Ning Jiang

**Affiliations:** 1 National Clinical Research Center for Geriatrics, West China Hospital Sichuan University, Chengdu, Sichuan Province China; 2 Department of Systems Design Engineering, Faculty of Engineering, University of Waterloo, Waterloo, Canada; 3 Med-X Center for Manufacturing, Sichuan University, Chengdu, Sichuan People’s Republic of China

**Keywords:** Biomedical engineering, Translational research

## Abstract

Surface electromyography (sEMG) signals have been used for advanced prosthetics control, hand-gesture recognition (HGR), and more recently as a novel biometric trait. For these sEMG-based applications, the translation from laboratory research setting to real-life scenarios suffers from two major limitations: (1) a small subject pool, and (2) single-session data recordings, both of which prevents acceptable generalization ability. In this longitudinal database, forearm and wrist sEMG data were collected from 43 participants over three different days with long separation (Days 1, 8, and 29) while they performed static hand/wrist gestures. The objective of this dataset is to provide a comprehensive dataset for the development of robust machine learning algorithms of sEMG, for both HGR and biometric applications. We demonstrated the high quality of the current dataset by comparing with the Ninapro dataset. And we presented its usability for both HGR and biometric applications. Among other applications, the dataset can also be used for developing electrode-shift invariant generalized models, which can further bolster the development of wristband and forearm-bracelet sensors.

## Abstract (summary)

**Table:** 

Measurement(s)	muscle electrophysiology trait
Technology Type(s)	electromyography
Factor Type(s)	Electrode Location • Hand Gestures • Session
Sample Characteristic - Organism	Homo sapiens

## Background & Summary

Recent advances in machine learning techniques have enabled applications of hand gesture recognition (HGR) using surface electromyographic (sEMG) signals. This has further boosted the development of advanced prosthesis control systems for rehabilitation of upper limb amputees^1^, and recent industrial applications have emerged using HGR for human-machine interactions in industrial^2^ and consumer applications scenarios^3,4^. For these applications, the sEMG signals features are extracted and used as inputs for various machine learning techniques such as linear discriminant analysis (LDA)^5^, support vector machines (SVM)^6^ etc. for detecting hand gestures. More recently, advanced techniques such as deep neural networks (DNN) have achieved highly accurate classification performance even with simple architectures^7,8^.

However, extensive investigation has demonstrated sEMG-based HGR has poor cross-user transference performance^9^, suggesting a calibration-free and one-size-fits-all model for all users is still elusive, which suggests that sEMG signals inherently contains individual differences, *i.e*., biometric information. This has provided motivation for investigating the potential of sEMG as a biometric trait. Combined with the HGR property, it enables the user to set user-defined gestures as a password for enhanced security, which is not possible with other bio-signals such as electroencephalogram (EEG) and electrocardiograph (ECG). Our recent studies have provided a framework for the fusion of these codes and to facilitate such a dual-mode (password and biometrics) authentication system^10^.

Although, high performance of both HGR and biometric models has been previously reported, there always exists a gap between the real-world conditions and the laboratory settings, under which most of the current HGR and biometric research have been conducted conditions. It has been established in the literature, that in a multi-session protocol spreading across days, non-stationary factors including electrode shifts, skin and physical conditions will seriously affect the performance of an sEMG processing system^11^. As there are numerous such factors, experimentally controlling each of them would increase the number of trial repetitions exponentially and hence can be arduous. Nevertheless, a multi-day dataset with a sufficiently large subject pool is warranted for validating the effectiveness of sEMG applications such as HGR and biometrics.

Some open-access databases of multi-day sEMG recordings of forearm muscles are publicly available^12–16^. Two databases with large subject pool (>40) involved two days of data collection^14,15^. While one of them has as low as six channels^15^, some others utilized a high-density (HD) sEMG setup^12,13,16^. Only three studies involved more than two of data collection days, but the number of subjects was smaller (<11)^12,17,18^. Only one study with three days of data recording, had a sample size of 20 subject, with only signals from the forearm^19^. To explore the robustness and accuracy of HGR and biometrics, it is imperative to have a database with larger subject pool sizes, recorded across multiple days and comprising numerous gestures.

**Fig. 1:**
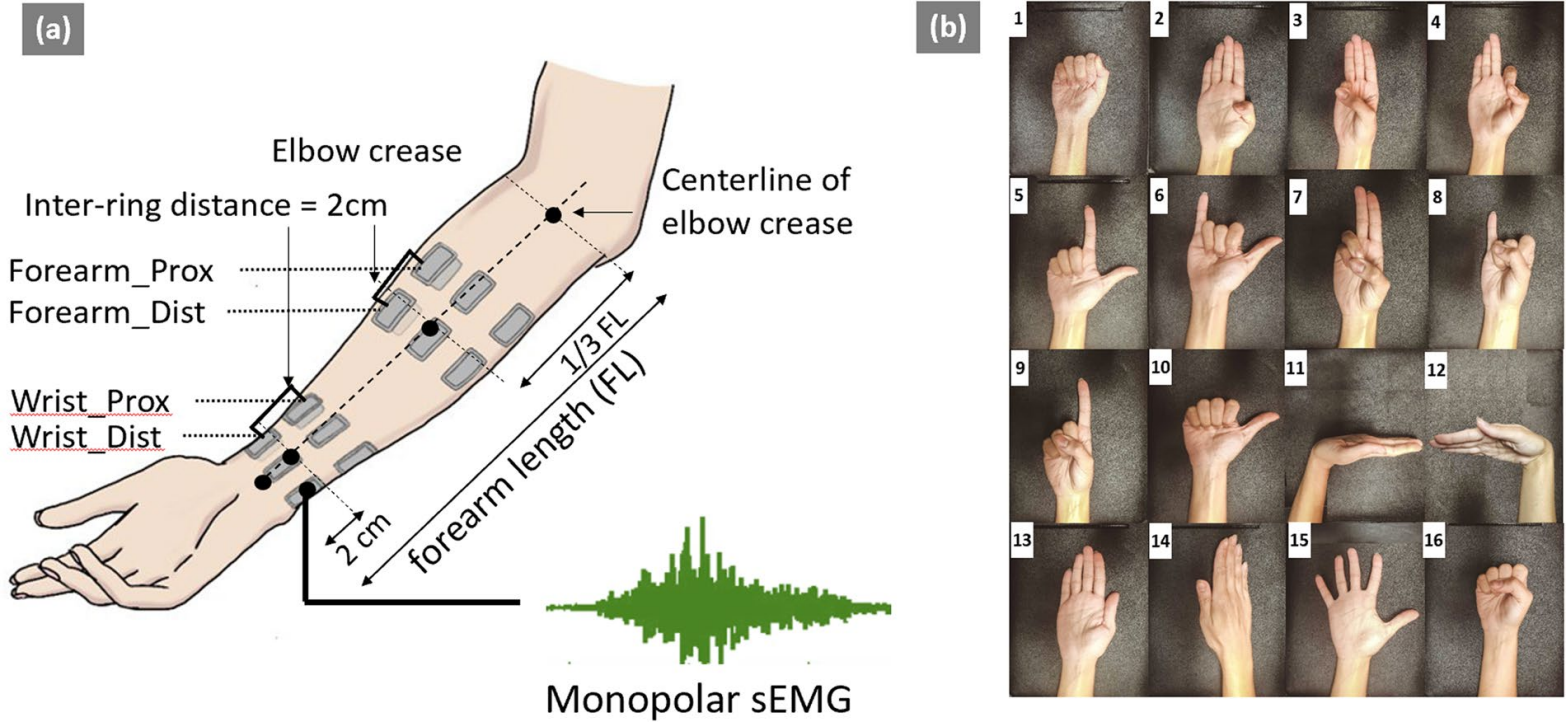
Electrode Positions and Gesture List. (**a**) (left) shows the electrode positions used in the study. There are two groups of electrodes: forearm and wrist. Each group comprises of two rings (proximal and distal). Monopolar sEMG was recorded from a total of 28 channels (16 Forearm and 12 wrist). (**b**) (right) shows the Sixteen gesture classes investigated in the study (numbered 1–16): lateral prehension (LP), thumb adduction (TA), thumb and little finger opposition (TLFO), thumb and index finger opposition (TIFO), thumb and little finger extension (TLFE), thumb and index finger extension (TIFE), index and middle finger extension (IMFE), little finger extension (LFE), index finger extension (IFE), thumb extension (TE), wrist flexion (WF), wrist extension (WE), forearm supination (FS), forearm pronation (FP), hand open (HO), and hand close (HC). The REST gesture was also collected in each trial repetition.

In the current study, we present an open-access named Gesture Recognition and Biometrics electroMyogram (GrabMyo) Dataset^20,21^. GrabMyo consists of 43 participants (subsequently termed as users), three sessions in three separate days (subsequently termed as sessions) of data collection, 16 hand and finger gestures each with seven repetitions (subsequently termed as trials). To our best knowledge, the presented dataset is the largest sEMG dataset in terms of the total number of recording sessions (43 users × 3 days = 129 recording sessions). A unique feature of GrabMyo is that the sEMG signals were recorded from both forearm and wrist positions. A graphical representation of electrode positions and the list of gestures investigated in the study is shown in Fig. 1a,b. The sampling frequency of the recorded signals was selected as 2048 Hz. To obtain generalizable data, special effort was taken such as 1) electrode positioning protocol for each session, 2) normal level force instruction, 3) rest duration for avoiding fatigue, 4) un-uniform interval between data collection sessions (Days 1, 8, and 29) and 5) data collection from healthy users (subjects with a single session of sickness have been eliminated from the study). These considerations are explained in greater detail in the Methods section. The dataset provides a valuable resource for sEMG-based HGR and biometrics research, particularly for improving algorithms’ robustness in a multi-day scenario and cross-user generalization ability.

## Methods

### Subjects and ethical requirements

We recruited 43 healthy participants (23 M, 20 F) for the study spanning three different days: day 1, day 8 and day 29. The participants were students and staffs from the University of Waterloo. The average age was 26.35 ± 2.89, and the average forearm length (measured from the styloid process on the wrist to the olecranon on the elbow) was 25.15 ± 1.74 cm. More details about the dataset and the participant characteristics are reported in Table 1. Individuals with any existing muscle pain, skin allergies, and those who were unable to complete the three sessions due to any unprecedent circumstances were excluded from the study. For the third session (day 29), a range of 3 days was provided for some participants who couldn’t make it to the specific appointment. Before the enrolment, the participants were provided an oral and written explanation of the procedures and signed an informed consent form. They were informed that they could withdraw from the study at any point. The experiments were conducted following the Declaration of Helsinki and the research protocol was approved by the Office of Research Ethics of the University of Waterloo (ORE# 31346).

**Table 1:** Database Summary.

Participant Characteristics	Values
# Males (# Females)	23 (20)
# Right-handed (#Left)	38 (5)
Age (years)	26.35 ± 2.89
Forearm length (cm)	25.15 ± 1.74
Forearm circumference (cm)	24.10 ± 2.27
Wrist circumference (cm)	16.18 ± 1.21

The forearm length is measured from the olecranon process to the ulnar styloid. The forearm circumference is measured at a distance one-third from the elbow joint. The wrist circumference is measured 2 cms away from the ulnar styloid process.

### Acquisition setup

The experimental setup consisted of a PC and a monitor mounted on a desk, 0.75 m in front of a height-adjustable chair. The EMGUSB2 + (OT Bioelettronica, Italy), a commercial amplifier, was used for acquiring the sEMG signals. The gain of the device was set to 500, and the sampling rate was set to 2048 Hz. Pre-gelled skin-adhesive monopolar sEMG electrodes (AM-N00S/E, Ambu, Denmark) were used.

Prior to the experiment, the user’s forearm length is measured as the distance between the olecranon process and the ulnar styloid process. The forearm circumference is measured at one-third of the forearm length from the olecranon process. The wrist circumference is measured at 2 cm away from the ulnar styloid process. Prior to electrode placement, the skin surface was shaved to remove hairs, cleaned with an alcohol swab, and abraded with a paper towel. For the forearm electrode placement, sixteen sEMG electrodes were placed in the form of two rings, each consisting of eight electrodes equally spaced around the forearm, forming eight bipolar pairs. The center-to-center distance between the two forearm-rings was maintained at 2 cm. For the wrist electrode setup, twelve monopolar sEMG electrodes of the same type as the forearm rings were placed in the form of two rings, each consisting of six electrodes equally spaced around the wrist and forming six bipolar pairs. The center-to-center distance between the two wrist-rings was maintained at 2 cms, the same as the forearm setup. Therefore, a total of 28 monopolar sEMG electrodes were used for each session, which forms four electrode rings: proximal and distal rings for the forearm and wrist. A detailed pictorial representation is provided in Fig. 1a. To maintain consistency of the positions of the electrodes across all participants, the first electrode in each ring (total rings = 4) was anatomically positioned on the center-line of the elbow crease as shown in Fig. 1a^22,23^. Although a standardized electrode placement protocol was followed for each session, no marks were left on the participant’s skin. This intentionally induced uncertainty in the exact electrode positions corresponds to the real-life scenario where it is not realistic, if possible at all, to place an electrode band at the same position across multiple sessions.

### Acquisition protocol

For each experimental session, following the acquisition setup, the participant is seated comfortably on the chair with both their upper limbs in a resting position. Visual instructions for performing the gestures were provided on the computer screen placed in front of the participants. The participants were instructed to perform the gestures at a normal force level, or similar to how they would normally do it during daily activities. For an estimate of “normal force”, the participants were asked to perform multiple trial contractions at three self-defined force levels: soft, hard and medium contractions, where a medium level corresponds to the contraction of the normal force. The following 16 hand and wrist gestures were included in the current study (presented in Fig. 1b): Lateral prehension (LP), thumb adduction (TA), thumb and little finger opposition (TLFO), thumb and index finger opposition (TIFO), thumb and little finger extension (TLFE), thumb and index finger extension (TIFE), index and middle finger extension (IMFE), little finger extension (LFE), index finger extension (IFE), thumb extension (TE), wrist flexion (WF), wrist extension (WE), forearm supination (FS), forearm pronation (FP), hand open (HO), and hand close (HC). The order of the 16 gestures was randomized and a resting (REST) trial was collected after all 16 gestures were performed once. A ten-seconds relaxing period was provided between each trial. One continuous data acquisition of 17 gestures (including the REST) is called one run. First, a trial run was performed to ensure that the participants understood the experimental protocol. Following the trial run, seven runs were recorded for each user, resulting in 119 contractions (17 × 7). Any accidental gesture or no-activity/delayed-activity was noted and the respective gesture’s replacement contraction were performed after each run. The user could also request additional rest when he/she felt necessary. The entire session was repeated on day 8 (after 1 week) and day 29 (after 1 month).

### Signal processing

The sEMG signals were bandpass filtered between 10 Hz and 500 Hz using a fourth-order Butterworth filter. A notch filter of 60 Hz was employed to remove the powerline noise that might have affected the signal recording.

## Data Records

Data records^20,21^ presented in this section and accompanying description files are available online in PhysioNet (https://physionet.org/about/database/) and IEEE Dataport (https://ieee-dataport.org/datasets). The database consists of 43 participants, three sessions in three separate days (subsequently termed as sessions) of data collection, and 17 gestures (including REST) each with seven repetitions. All the sEMG recordings are 5 seconds in duration, collected from 28 channels (16 forearm and 12 wrist), and sampled at 2048 Hz.

The components of the released repositories are described in detail (Table 2) and are organized as follows: waveform data and additional files.

**Table 2:** Multi-Day EMG Database.

Repository	Folders	Sub-Folders	Files	Description
PhysioNet	Session 1	session***i***_subject***j***	session***i***_subject***j***_gesture***k_***trial***l****.*dat	𝒊 ∈ [1,2,3] represents the session(day) index
	Session 2		session***i***_subject***j***_gesture***k_***trial***l****.*hea	***j*** ∈ [1, 2,…43] represents the subject index
			(.dat file contains 10240 × 32 hexadecimal values.	
	Session 3		.hea file contains signal information such as sampling frequency, units and gain)	*k* ∈ [1, 2,…17] represents the gesture index
				*l* ∈ [1, 2,…7] represents the trial index
IEEE Dataport	Session 1	subject***i***_session***j***	session***i***_subject***j****.*mat (.mat file contains 17 × 7 cell matrix. each cell contains 10240 × 32 numeric array)	𝒊 ∈ [1, 2, 3] represents the session(day) index
	Session 2			***j*** ∈ [1, 2,…43] represents the subject index
	Session 3			
Additional Files				
PhysioNet	<N/A>	<N/A>	readme.txt, Subject-info.csv, MotionSequence.txt, GestureList.jpg, DeviceInfo.pdf, Electrodelocation.pdf	*readme.txt* general information of the dataset and reading data files.
				*MotionSequence.txt* provides gesture definitions and their sequence
				*GestureList.jpg* provides pictorial representation of hand gestures.
IEEE Dataport	<N/A>	<N/A>	Subject-info.csv, MotionSequence.txt, GestureList.jpg	*DeviceInfo.pdf* describes the configuration of acquisition device.
				*ElectrodeLocation.pdf* illustrates the positioning of electrode sensors.
				*Subject-info.csv* contains participant anthropometric information

MATLAB Codes: File Reading and Signal Processing (explained in Code availability section).

For the physionet.org repository, the signal files are converted to the waveform database (WFDB) format (a *.dat file containing the signed 16 bit quantized value and *.hea file with the same name containing the scaling factors. For the IEEE Dataport *.mat file with same name is generated which consists of multiple recordings from trials and gestures.

### Waveform data

For the PhysioNet Database, the sEMG recordings are provided in the waveform database (WFDB) format, which is considered the most widely used medium for storing physiological signals and waveform data^24^. There exist numerous open-source WFDB libraries for commonly used analysis tools using MATLAB and Python. The WFDB allows a structured way of storing the sEMG recording in the form of a tuple of two files: a dat-file containing the binary raw data and a corresponding header file with the same name and a hea-extension. The hea-extension contains all the signal-specific metadata such as the channel names, sampling frequency, and the scaling factor for converting the signal to physical units (in mVs).

For the IEEE database, the signal files are saved as .*mat* files. The mat file extension allows users a convenient approach for obtaining the sEMG recordings in the physical unit format (additional conversion is not necessary). Additionally, the mat files allow data to be organized as cells where multiple gestures and repetitions from a single session can be presented together.

### Additional files

The additional files provide supplementary information such as the participant anthropometrics, electrode positioning guidelines, device configuration, the sequence of gestures, and their descriptions. MATLAB codes are also provided for reading the data files and subsequent feature extraction as explained in the Code Availability section.

## Technical Validation

**Fig. 2:**
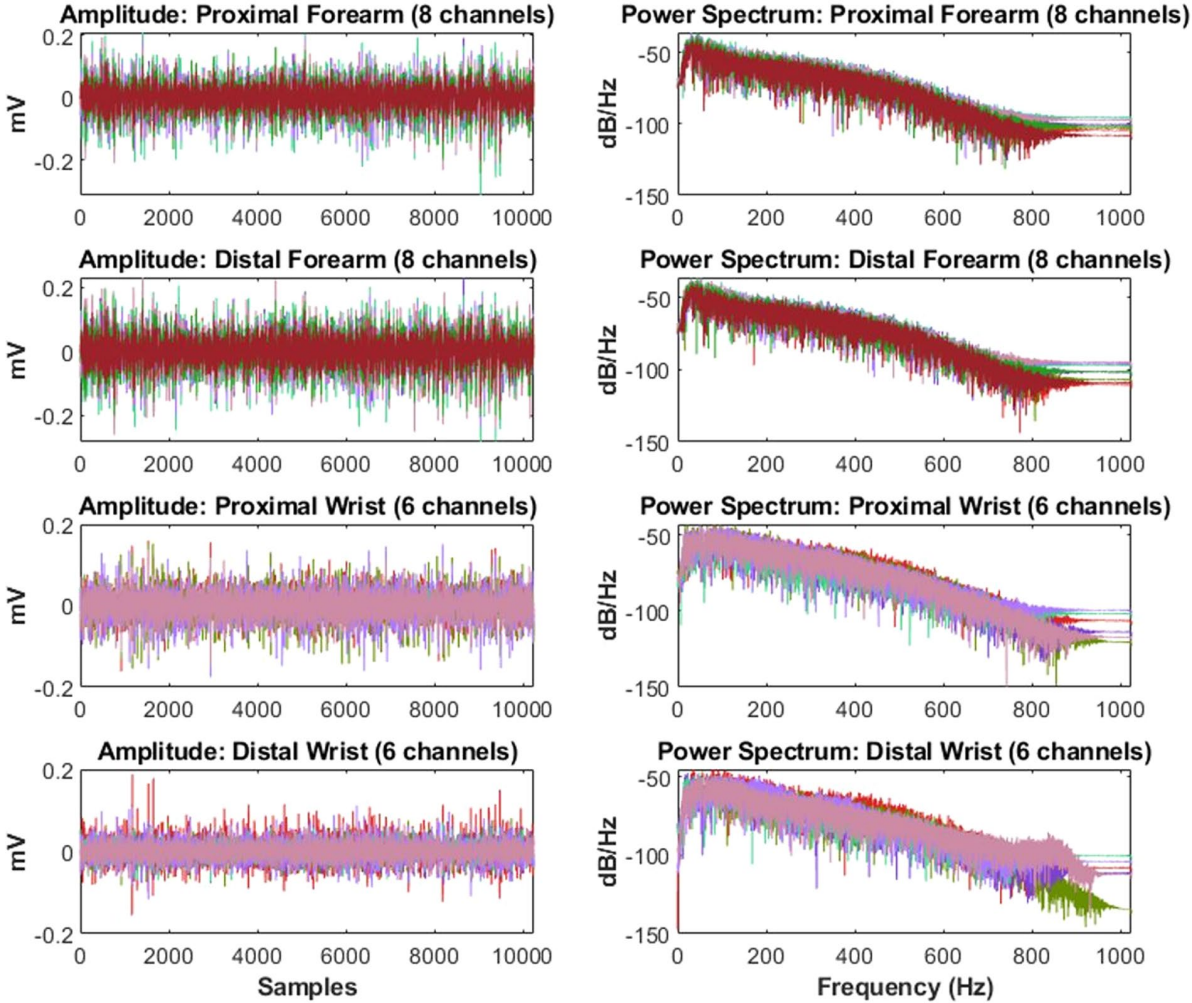
Representative amplitude and power spectum plots. Left Column: The amplitude values (in mV) for the four electrode rings: proximal and distal forearm and wrist (from top to bottom). Right Column: The fast fourier transform (FFT) power spectrum (in dB/Hz) for the four electrode rings: proximal and distal forearm and wrist (from top to bottom). The forearm rings constitute 8 channels each and the wrist rings constitute 6 channels each (shown in different colors).

A specific trial of an individual user case was chosen at random and the amplitude and frequency components were analyzed as shown in Fig. 2. It was observed that all 28 channels of forearm and wrist possessed equal amplitude and power spectrum distribution. In the following sections, signal processing techniques are discussed for analysis and technical validation of the sEMG signals. As well, the original references where the methods were described in depth are provided. For comparison, the validation results are provided along with those for the widely used Ninapro DB2: Exercise B comprising 40 users and 17 gestures.

### Signal-to-noise ratio (SNR)

**Fig. 3:**
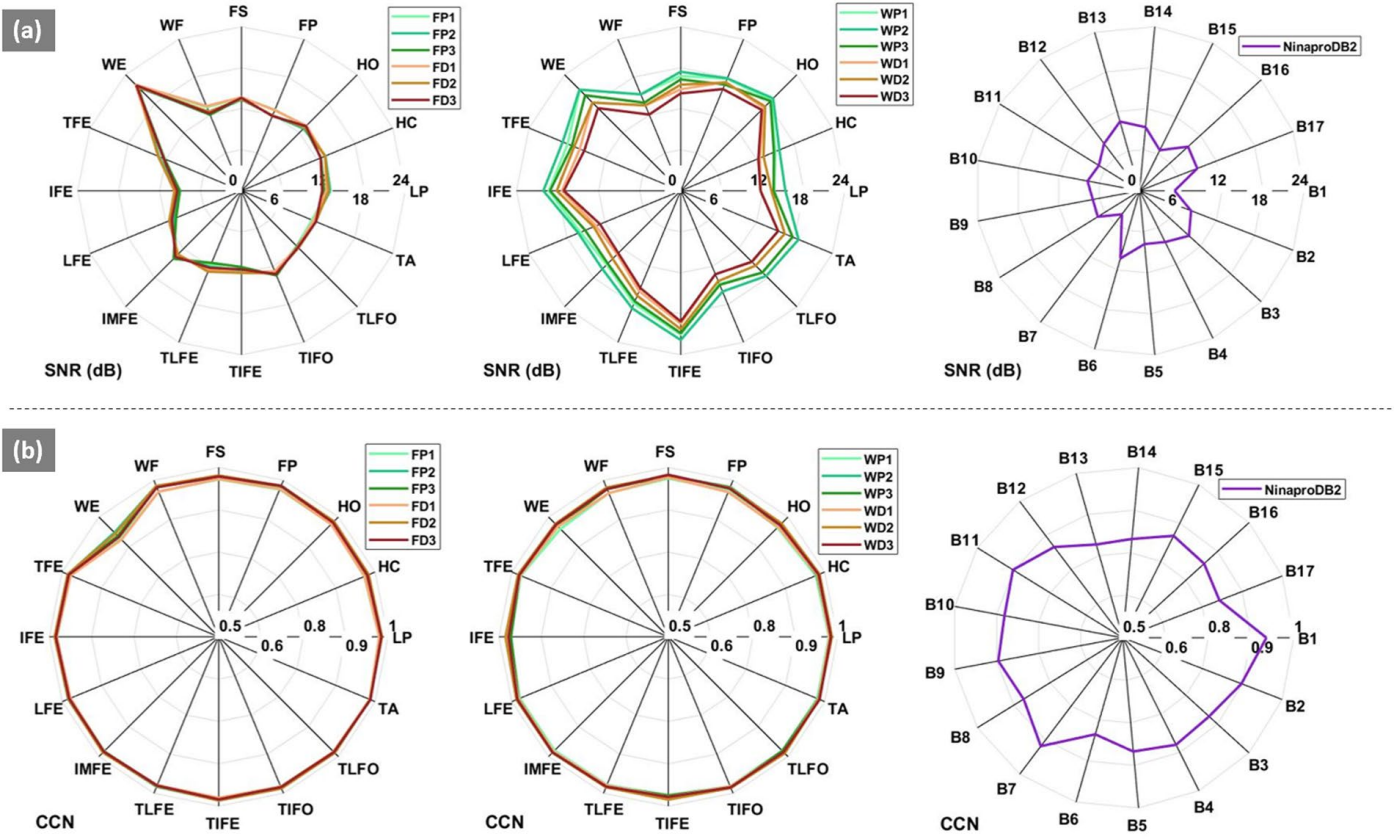
SNR and CCN comparison plots. Panel **a**, *i.e*. the top row shows SNR values (dB), with each spoke representing one motion. Left: the forearm proximal and distal electrode setup for session 1–3 (added as suffix FP1, FP2,…,FD3); middle: wrist proximal and distal electrode setup for session 1–3 (added as suffix WP1,WP2,…,WD3); and (right) the Ninapro signals collected from the forearm for a single day. Panel **b**, *i.e*. the bottom row shows the CCN values for the forearm (left), wrist (middle) and Ninapro data (right) in a similar manner.

The spectral properties of each sEMG signal recording were analysed and the SNR (in dB) was measured as the ratio between the power of the signal to the power of the noise^25,26^. As the types of the artifacts were unknown in our case, the power of the noise was estimated as the power of sEMG recordings during the rest trial. The average SNR of all the signals (14.565 ± 6.385 dB) was in range with the SNR values suggested for the wrist and forearm^25^ and they are consistently higher than those from the Ninapro database (8.001 ± 4.051 dB) (see Fig. 3a).

### Correlation coefficient of Normality (CCN)

The CCN was measured to analyze the amplitude distribution. For a static contraction with moderate force, the sEMG can be modeled as a filtered, random, white Gaussian noise process^27^. It has been suggested that a test of normality can provide a measure of biosignal quality, where a signal amplitude with a non-normal distribution would be considered contaminated. A Gaussian distribution with equal mean and variance to that of the recording is generated^28^. The CCN is defined as the Pearson correlation coefficient between the histogram bin values of the sEMG recording and the normal density function value for the corresponding bins. A value close to 1 is considered a normal distribution. It was observed that the CCN of all the signals (0.975 ± 0.041) was close to 1 which was consistently higher than those from the Ninapro database (0.848 ± 0.075) (see Fig. 3b).

### Standard HGR and biometric analysis

Prior to HGR and Biometric evaluation, the signals were first processed and then features were extracted as described below. The forearm rings (eight channels) and the wrist rings (six channels), the monopolar sEMG signals were first re-referenced by a common average procedure. The processed signals were then segmented into 200 ms-width windows, with a 150 ms overlap. Each window was then processed using Hudgins’s time-domain (TD) feature extraction^29^. Time-domain features (mean absolute value, zero crossing, slope sign changes, and waveform length) were extracted from filtered data^22^. Therefore, for a forearm setup, the feature vector consisted of 8 × 4 = 32 features, while the wrist setup consisted of 6 × 4 = 24 features.

For the biometric analysis, a matching score, commonly the Mahalanobis distance, is used to assess if it’s a match (access granted) or no match (access denied)^10,22,23^. To maintain consistency in the HGR and biometric analyses, a Mahalanobis distance classifier was implemented for both analyses. For a given feature vector sample *p* (the input), its Mahalanobis distance *S*_*i.j*_, with the *i*th gesture and the *j*th user, was defined as

1 $${S}_{i,j}\left(p\right)=\sqrt{{\left(p-{{\rm{\mu }}}_{i,j}\right)}^{{\rm{T}}}{\Sigma }_{i,j}^{-1}\left(p-{{\rm{\mu }}}_{i,j}\right)},$$

where *µ*_*i,j*_ is the centroid of the *i*th gesture class and the *j*th user and ∑_*i, j*_ is the covariance matrix for the specific gesture and user class. Both the parameters are calculated from the system training data and the sample *p* is from the system testing data. The leave-one-out (LOO) cross-validation scheme was used, where six trials were used for training and one trial for testing. Figure 3a,b, and Table 3 demonstrate the results of the technical validation as described in the following sections.

**Table 3:** Mean (±STD) HGR and Biometric performance.

Electrode Setup	HGR Evaluation (AUC)	Biometric Evaluation (EER)
Forearm	0.948 (±0.018)	0.028 (±0.007)
Wrist	0.941 (±0.021)	0.038 (±0.006)
Ninapro (Forearm)	0.875 (±0.034)	0.038 (±0.013)

#### HGR evaluation

**Fig. 4:**
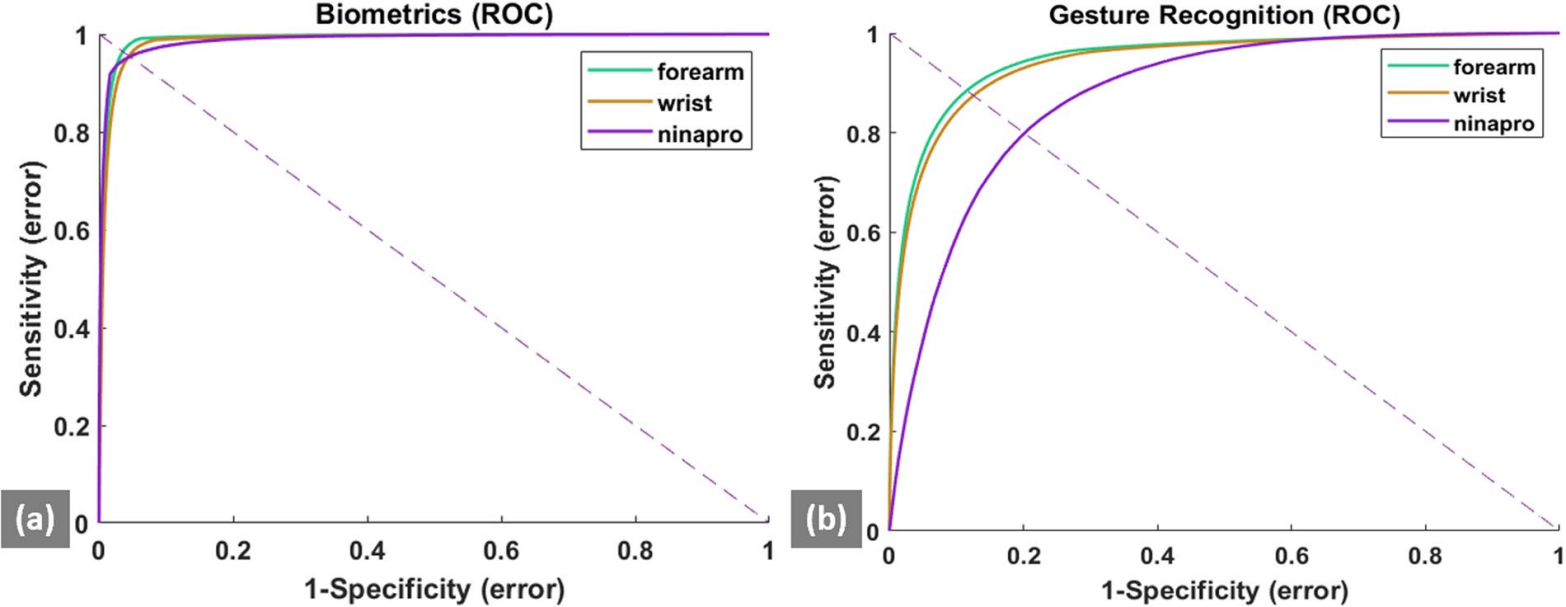
ROC curves for HGR and Biometric validation. The ROC curves are plotted for the HGR evaluation (**a**) and Biometric Evaluation (**b**) for the forearm and wrist data averaged for the multiple users (N = 43), sessions (N = 3) as well as for the Ninapro Data (#users = 40, #sessions = 1). The dotted line represents the point of intersection on the ROC curve from which the EER value is obtained.

In this study, the HGR analysis was performed in a user-specific scheme. For a particular user and a particular gesture, the true class consisted of the feature vectors from the target gesture of the user and the false class consisted of the feature vectors from the remaining 15 gestures of that user (the rest gesture was excluded from the HGR analysis to maintain consistency with biometric analysis). Similarly, for the Ninapro database, the true class consisted of feature vectors from the target gesture of a specific user and the false class consisted of the feature vectors of the remaining 16 gestures. For the performance analysis a receiver operating characteristic (ROC), where the true positive rate (sensitivity) was plotted against the false positive rate (1 – specificity) by varying the threshold distance of correct gesture prediction. The true positive rate or sensitivity represents the probability of detecting a correct gesture, while the false positive rate is the probability of detecting an incorrect gesture. The area under the curve (AUC) is calculated from the ROC curve^30^. The ROC curve and the AUC values for all the users, days, and gestures are averaged and reported separately for the forearm and wrist electrode positions. Figure 4a shows the ROC plots for HGR analysis. It was observed that the AUC for the forearm was 0.948 (±0.018) and for the wrist was 0.941 (±0.021). Both the values were comparatively higher than the corresponding AUC value of 0.875 (±0.034) for the NinaPro data.

#### Biometric evaluation

In this study, the verification mode of biometrics was used to demonstrate the feasibility of multi-day sEMG-based biometrics. In this mode, the true user’s identity and the corresponding gesture are known to the system. As such, for a specific user, the true class consisted of the feature vectors from the target gesture (*e.g*. the passcode) of that user and the false class consisted of the feature vectors of the remaining 42 users for that gesture. Similarly, for the Ninapro database, the true class consisted of feature vectors from the target gesture of a specific user and the false class consisted of the feature vectors from the remaining 39 users for that gesture. For the performance analysis a receiver operating characteristic (ROC), where the true positive rate (sensitivity) was plotted against the false positive rate (1 – specificity) by varying the threshold distance of correct biometric authentication. The true positive rate or sensitivity represents the probability of detecting a correct hand gesture, while the false positive rate is the probability of detecting an incorrect hand gesture. The equal error rate (EER), is obtained from the ROC curve, where the false positive rate is equal to the false negative rate (1– sensitivity)^30^. The ROC curve and the EER values for all the users, days, and gestures are averaged and reported separately for the forearm and wrist electrode positions. Figure 4b shows the ROC plots for Biometric analysis. It was observed that the EER for the forearm was 0.028 (±0.007) and for the wrist was 0.038 (±0.006). Both the values were comparatively lower than the corresponding EER value of 0.038 (±0.013) for the NinaPro data.

## Usage Notes

As evident from the title, the GRABMyo Dataset has two major applications: HGR using machine learning approaches and sEMG-based biometrics. The true potential of GRABMyo are three-fold: 1) large subject-pool, 2) multi-day session, and 3) both wrist and forearm channels. Through the *Signal Analysis* section, we will explain processing sEMG recordings and the cross-validation approach for obtaining reliable benchmark results using machine learning algorithms. Following this, the *Future Direction* lists novel research strategies for HGR and Biometric analysis which could be bolstered using the large multiday dataset.

### Signal analysis

The MATLAB code fileread.m allows the users to read data files along with their signal-specific metadata. After conversion to a numeric format, the data is structured as matrixes with timepoints as rows and monopolar sEMG channels as columns. The channel names provided can be used for separate forearm and wrist electrode rings and also for forming bipolar pairs between them^31^. The processed data is then windowed, and features can be extracted using multiple techniques^32^. A widely used frequency division technique^33^ is provided as a sample feature extraction in feature_extraction.m. The gesture labels are provided by MotionSequence.txt which allows the extracted features to be matched to the specific gestures and their respective users. For HGR and biometric applications, the output classes are the gestures and users, respectively which can be used for developing machine learning and deep learning architectures. The data provides seven trial repetitions, hence seven-fold cross-validation (six-folds for training and one-fold for testing) can be used to simulate a near-practical scenario, where the testing data is recorded separately from the testing data. A more robust analysis *i.e*., the multi-day analysis with a 3-fold cross-validation can be employed, where subsets of data from two days are used for training and the data from the remaining day is used for testing.

### Future Directions

Some future research directions are:

#### Improving biometric authentication

One unique advantage of the EMG biometric trait, in particular for authentication applications, is the combination of user-specific biometrics with user-defined gestures as passcodes, the latter of which is not possible with other bio-signals such as electroencephalogram (EEG) and electrocardiogram (ECG). A multi-code EMG-based biometric framework can be used to combine the gestures and improve authentication performance and security^10^.

#### Biometric identification

Another major biometric application is the identification mode where the system predicts the identity of the presenting user by finding the closest match. The identification is a more error-prone application as the system makes *N* comparisons, where *N* is the number of users. Therefore, the factors affecting system performance such as multiple days and sample size of the database need to be investigated for real-life applications.

#### Subject independent gesture recognition

Extensive research on EMG has been performed on gesture recognition with application in rehabilitation using prosthetic and orthotic devices, home application for assisting daily activities, virtual environment control, and sign language recognition^3,4,34^. Recent studies have suggested deep learning techniques for cross-user calibration-free which trains generalized models using the population data, and hence reduces the training burden of the user^9,35,36^. The presented large-sample dataset can provide resources for such calibration-free models.

#### Electrode shift-invariant techniques

One of the significant factors affecting the cross-day sEMG performance is the shift in the electrode positions. It is impossible to fix the location of armband electrodes on the forearm and wrist for daily-wear use. These variations affect the performance of both the sEMG-based biometric and gesture recognition applications. Some techniques such as classification model adaptation^37,38^ and feature space transformation using transfer learning^35,39,40^ have been suggested to address the electrode shift variations. These techniques could be further investigated to potentially improve biometrics and HGR performance.

## Data Availability

The custom codes used for reading the signals of the database was created in MATLAB R2017b and is freely accessible at Physionet and IEEE Dataport^20,21^. To implement the codes, the users will need a MATLAB License • A readme file (readme.txt) with instructions about how to run the code in a 2017b or higher MATLAB version. • A Matlab script (fileread.m)with a simple example about how to read WFDB files and convert them to.mat format. • A text file(MotionSequence.txt)which provides the gesture sequence, and thus can be used to assign class labels to input data. • A Matlab script(feature_extraction.m)allows a simple example to extract frequency features using featiDFTI.m and segmentEMG.m functions. • A Matlab function(featiDFTI.m)for generating frequency division technqiue features from sEMG Data. • A Matlab script(segmentEMG.m)for implementing windowing of sEMG Data.
